# Combination of serum and CSF neurofilament-light and neuroinflammatory biomarkers to evaluate ALS

**DOI:** 10.1038/s41598-020-80370-6

**Published:** 2021-01-12

**Authors:** Alexandre Brodovitch, José Boucraut, Emilien Delmont, Amandine Parlanti, Aude-Marie Grapperon, Shahram Attarian, Annie Verschueren

**Affiliations:** 1 Timone Hospital, Referral Centre for Neuromuscular Diseases and ALS, AP-HM, Marseille, France; 2 Immunology Laboratory, Conception Hospital, AP-HM, Marseille, France; 3 INT UMR CNRS 7289, Medicine Faculty, Aix Marseille University, 27 Boulevard Jean Moulin, 13385 Marseille Cedex 05, France; 4 CRMBM UMR CNRS 7339, Aix Marseille University, Marseille, France; 5 INSERM UMR S910, GMGF, Aix Marseille University, Marseille, France

**Keywords:** Biomarkers, Neurology

## Abstract

This monocentric prospective study of patient suffering from Amyotrophic lateral sclerosis (ALS) aims to evaluate the prognosis and diagnostic potential of both Neurofilament-Light (Nf-L) and neuroinflammatory biomarkers in serum and CSF. Candidate markers levels were measured using multiplex method in serum of 60 ALS patients, 94 healthy controls of 43 patients suffering from Inflammatory Peripheral Neuropathies (IPN). A comparative CSF analysis was performed for 20 ALS and 17 IPN patients. Among the altered biomarkers, CSF Nf-L level remains the best marker of ALS severity, while serum levels correlate strongly with disease progression. The combination of Nf-L and ICAM-1 concentrations in the CSF and IFN-γ concentration in the serum differentiate ALS patients from IPN patients with improved sensibility and specificity relative to individual biomarkers. A cutoff value of 0.49 for the fitted values of these 3 biomarkers discriminate ALS from IPN patients with a specificity of 100% (78.20–100%) and a sensibility of 85.71% (57.19–98.22%) with an AUC of 0.99 ± 0.01. The measure of Nf-L and neuroinflammatory biomarkers in CSF and serum can be useful biomarkers panel in the differential diagnosis of ALS.

## Introduction

Amyotrophic lateral sclerosis (ALS) is a neurodegenerative disease characterized by a great variability of clinical presentations and prognosis between patients. Moreover, misdiagnosis of ALS remains a common clinical problem at the onset phase^1^ especially since certain pathologies can benefit from effective treatment^2^. It is therefore challenging to identify a biomarker or a combination of biomarkers to assist in the management and monitoring of this pathology^3,4^.

The most advanced biomarker for ALS is Neurofilament Light chain (Nf-L)^5–7^. The major interest for this marker is that serum as well as cerebrospinal fluid (CSF) levels correlate with disease progression and survival rates. However, this marker is not specific to the pathology and is discussed in other neurodegenereative diseases or peripheral neuropathies^8–10^.

Neuroinflammation is a hallmark of many central and peripheral nervous diseases. These neuroimmune processes can either participate in neurodegeneration or exert a neuroprotective effect^11^. ALS is associated with immune cell recruitment, glial cell inflammation and changes in immune parameters in the CSF and the blood^12,13^.Several studies reported increased levels of several blood and CSF biomarkers, including vascular biomarkers (ICAMs, VCAMs, VEGF) chemokines (MCP-1, Eotaxin) and cytokines (IL-6, IL-17, IL-8, IL-10, IFN-γ, TNF-α)^4,14^. Based on these data from the literature, and the possibility of using multiplex and high-sensitivity techniques with little matrix effect, we evaluate the clinical interest of combining inflammatory biomarkers and Nf-L assays in the blood and CSF for ALS.

## Results

### Serum biomarker analysis

**Figure 1:**
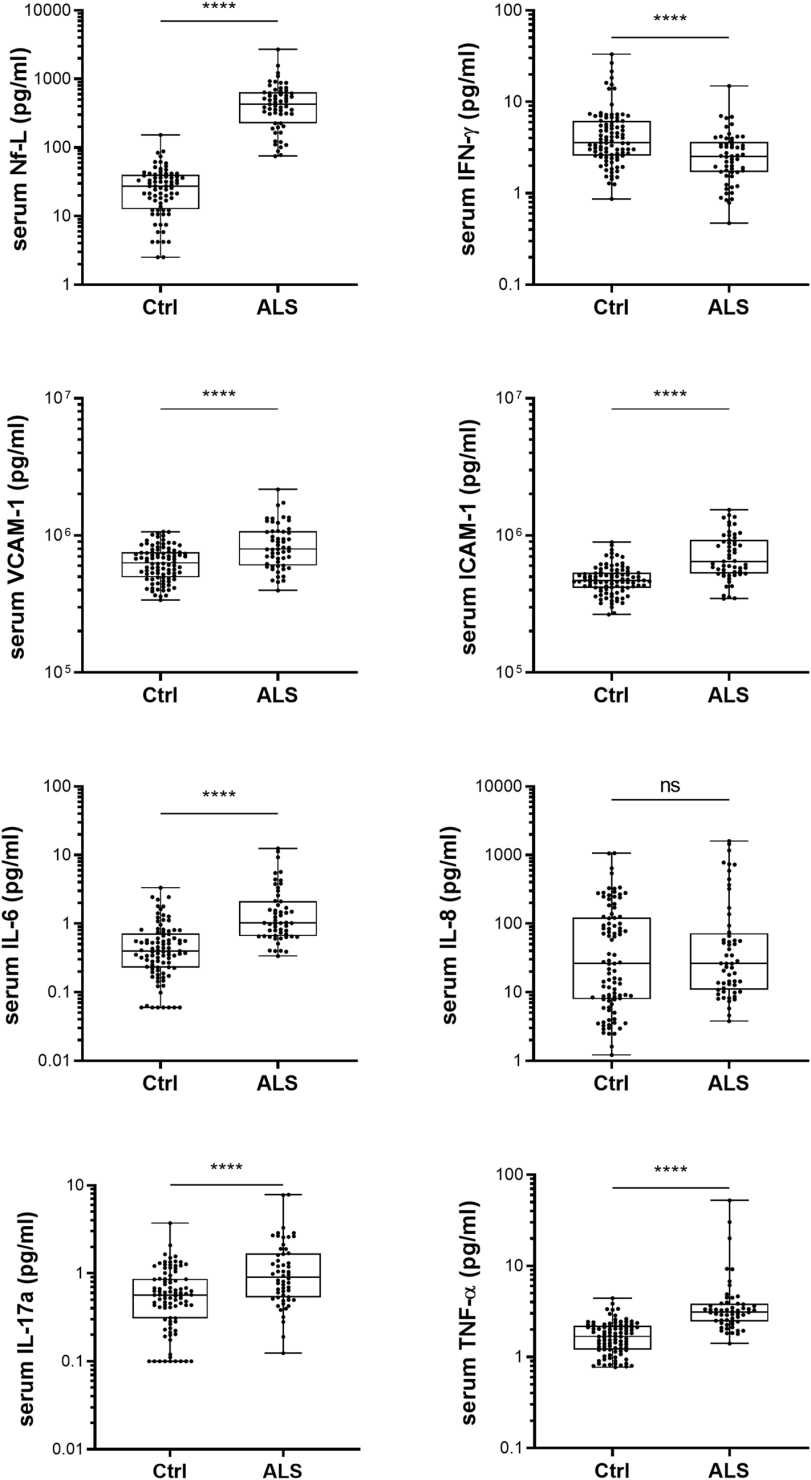
Neuroinflammation biomarkers in serum samples of ALS patients. Levels of biomarkers in serum from ALS patients (ALS) compared to serum sample from the control group (Ctrl). Mann Whitney test significance levels are indicated on graphs (****p < 0.0001).

Among the 15 analyzed factors in serum, 9 of the biomarkers were significantly elevated in the serum of 60 ALS patients compared to healthy controls (Fig. 1 and Table 1).

**Table 1:** Biomarkers levels in serum of patients with ALS and control group (Ctrl).

Serum	Ctrl	ALS	*p* value
	(n = 94)	(n = 60)	
Nf-L (pg/ml)	30.20 ± 23.41	512.4 ± 417.4	** < 0.0001**
VCAM-1 (ng/ml)	647 ± 181	891 ± 366	** < 0.0001**
ICAM-1 (ng/ml)	485 ± 120	750 ± 297	** < 0.0001**
VEGF (pg/ml)	199.1 ± 232.5	150.0 ± 77.23	0.523
Eotaxin (pg/ml)	242.4 ± 143.3	284.5 ± 104.6	0.134
MCP-1 (pg/ml)	256.9 ± 96.94	373.8 ± 169.1	** < 0.0001**
IP-10 (pg/ml)	384.7 ± 289.5	640.2 ± 320.4	** < 0.0001**
IL-17a (pg/ml)	0.68 ± 0.53	1.38 ± 1.48	** < 0.0001**
TNF-α (pg/ml)	1.76 ± 0.72	4.99 ± 7.85	** < 0.0001**
IL-2 (pg/ml)	N.A	0.40 ± 0.41	
IL-10 (pg/ml)	0.29 ± 0.34	0.51 ± 0.19	** < 0.0001**
IL-8 (pg/ml)	106.1 ± 186.5	172.3 ± 354.9	0.395
IL-6 (pg/ml)	0.58 ± 0.58	2.0 ± 2.6	** < 0.0001**
IL-1β (pg/ml)	N.A	0.47 ± 0.73	
IFN-γ (pg/ml)	5.31 ± 5.22	2.96 ± 2.25	** < 0.0001**

Significant Mann Whitney *p* values are indicated in bold.

Levels of Nf-L in serum are higher in the ALS sample compared to control group (512.4 ± 417.4 pg/ml in ALS vs. 30.20 ± 23.41 pg/ml in Ctrl; *p* < 0.0001). Inflammatory cytokines and chemokines (IL-6, IL-10, IL-17a, TNF-α, IP-10 and MCP-1) levels are significantly higher in the serum of ALS patients compared to controls. Levels of vascular markers VCAM-1 and ICAM-1 were increased in serum samples of ALS patients when compared to controls (891 ± 366 ng/ml vs. 647 ± 181 ng/ml; *p* < 0,0001 for VCAM-1 | 750 ± 297 ng/ml vs. 485 ± 120 ng/ml; *p* < 0,0001 for ICAM-1). Due to many values below threshold in the control group, variations IL-1β and IL-2 levels could not be analyzed between ALS and controls. Only IFN-γ decreases in the ALS group compared to the control group (Fig. 1; 2.96 ± 2.25 pg/ml in ALS vs. 5.31 ± 5.22 pg/ml in Ctrl; *p* < 0.0001).

No significant correlation between the neurodegeneration marker, Nf-L and each of the neuroinflammation markers was observed.

The control population includes 21 near-age patients from the ALS patient population. Biomarkers levels similar to those observed in the total population, confirming the presence of peripheral inflammatory processes in ALS patients. These processes are obviously non-specific to the pathology. Compared to the cohort of patients with inflammatory neuropathy, there is a very significant increase in Nf-L and an increase in VCAM-1, ICAM-1, IL-10 and IP-10. On the other hand, the decrease in IFN-γ is specifically associated with ALS (See Supplementary Table 1 and Supplementary Fig. S1 on line).

**Figure 2:**
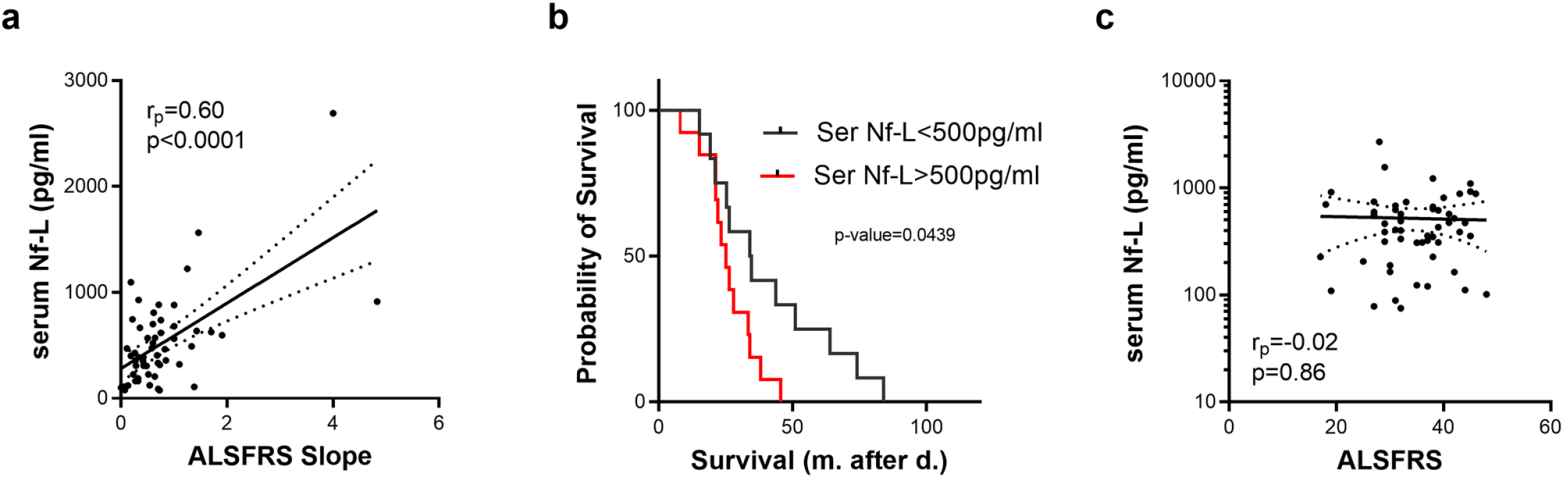
Serum Nf-L association with clinical parameters. Association between Nf-L concentration in serum and clinical parameters (**a**: ALSFRS Slope; **c**: ALSFRS). Kaplan–Meier survival curves of ALS patients according to serum (**b**) Nf-L levels.

The ALSFRS slope, indicative of disease progression rate, is correlated with serum Nf-L levels (Fig. 2a rp = 0.60; *p* < 0.0001). Furthermore, a serum Nf-L concentration above 500 pg/ml is associated with shorter survival (Fig. 2b). However, disease severity, evaluated by ALSFRS-R score (Fig. 2c), did not correlate with Nf-L levels whereas serum levels of MCP-1 (rp =  − 0.31; *p* < 0.05) and VEGF (rp =  − 0.31; *p* < 0.05) are correlated with ALSFRS-R score.

### CSF biomarkers analysis

**Figure 3:**
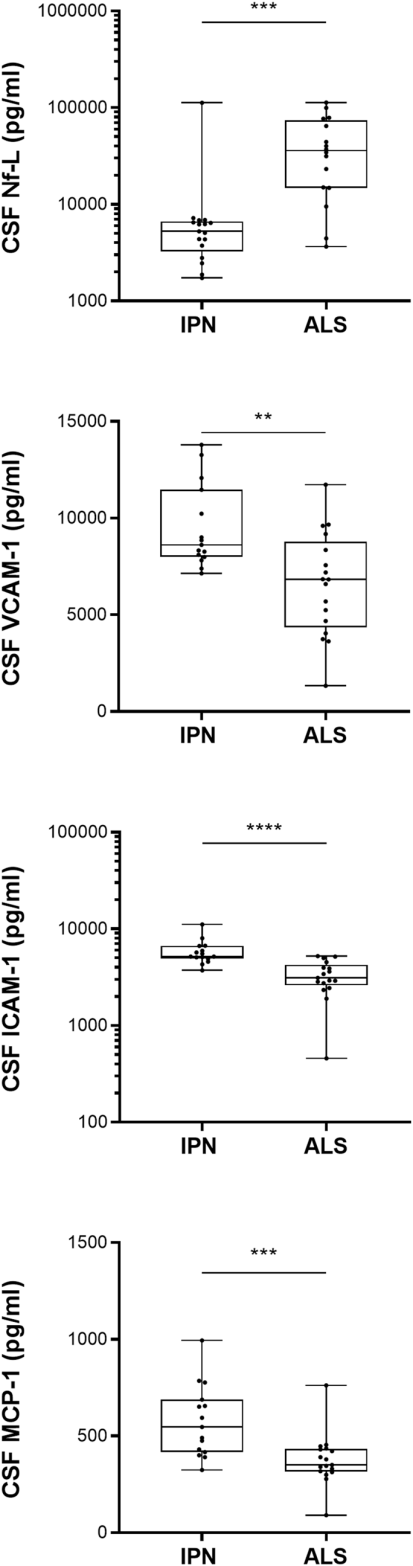
CSF markers in ALS and IPN. Difference in CSF markers between ALS and IPN patients. Mann Whitney tests significance levels are indicated on graphs (**p < 0.01; ***p < 0.001; ****p < 0.0001).

We compare 20 CSF biomarkers from ALS with CSF marker of 17 IPN patients. Results are detailed in the Table 2 and Fig. 3. As for serum analysis, the CSF Nf-L level is also sharply increased in ALS (43,257 ± 34,120 pg/ml vs. 11,196 ± 26,100 pg/ml; *p* = 0.001) when compared to IPN (Fig. 3).

**Table 2:** Biomarkers levels in CSF of patients with IPN or ALS.

CSF	IPN	ALS	*p* value
	(n = 17)	(n = 20)	
Nf-L (pg/ml)	11,196 ± 26,100	43,257 ± 34,120	**0.001**
VCAM-1 (ng/ml)	9.5 ± 2.2	6.6 ± 2.7	**0.004**
ICAM-1 (ng/ml)	5.9 ± 1.8	3.3 ± 1.3	** < 0.0001**
VEGF (pg/ml)	1.8 ± 1.4	2.7 ± 1.5	0.042
Eotaxin (pg/ml)	7.6 ± 4.6	8.1 ± 3.2	0.313
MCP-1 (pg/ml)	575.0 ± 184.7	375.8 ± 131.9	**0.001**
IP-10 (pg/ml)	334.9 ± 22.8	362.7 ± 227.5	0.823
IL-17a (pg/ml)	0.12 ± 0.11	0.19 ± 0.14	0.156
TNF-α (pg/ml)	0.25 ± 0.24	0.16 ± 0.11	0.079
IL-2 (pg/ml)	0.15 ± 0.07	0.38 ± 0.22	0.085
IL-10 (pg/ml)	0.15 ± 0.28	0.10 ± 0.06	0.634
IL-8 (pg/ml)	46.16 ± 60.17	34.48 ± 12.10	0.631
IL-6 (pg/ml)	1.6 ± 1.0	0.96 ± 0.47	0.063
IL-1β (pg/ml)	0.07 ± 0.06	0.36 ± 0.68	0.104
IFN-γ (pg/ml)	1.0 ± 2.8	0.53 ± 0.32	0.192

Mann Whitney test *p* values below 0.01 are indicated in bold.

Interestingly, while serum levels of VCAM-1 and ICAM-1 are higher in ALS than in IPN as previously reported (See Supplementary Fig. S1 on line), levels of VCAM-1, ICAM-1 and MCP-1 are significantly lower in the CSF of ALS compared to IPN (Table 2 and Fig. 3).

**Figure 4:**
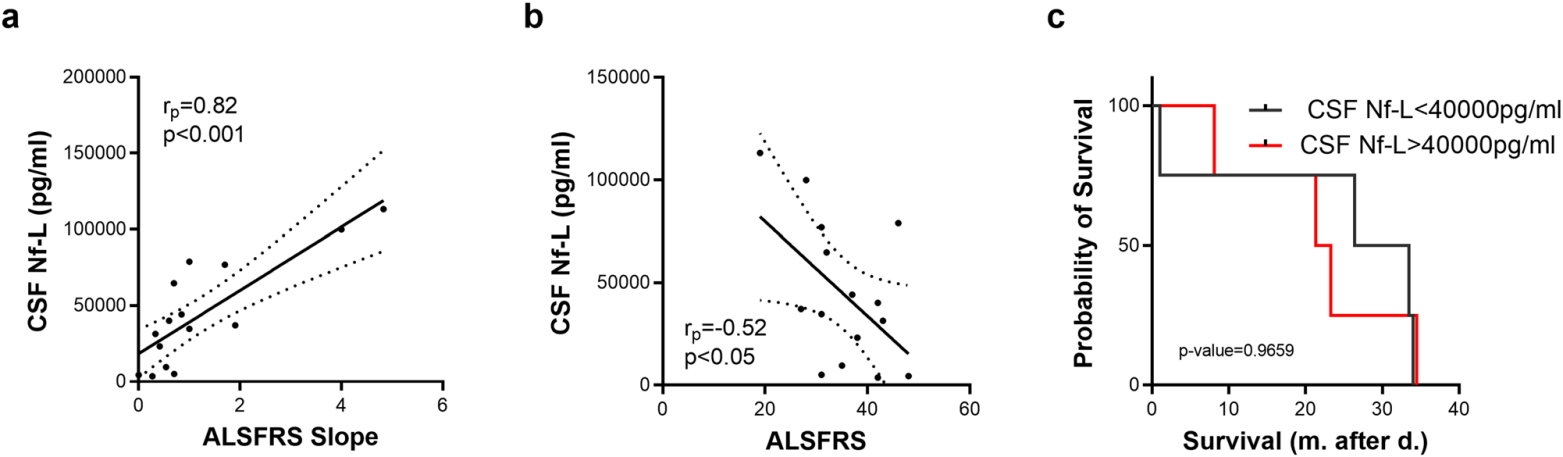
CSF Nf-L association with clinical parameters. Association between Nf-L concentration in CSF and clinical parameters. Kaplan–Meier survival curves of ALS patients according to CSF (**c**) Nf-L levels.

Disease severity, evaluated by ALSFRS-R score and the ALSFRS slope are both correlated with Nf-L levels in the CSF (Fig. 4a,b).) Thus, despite a good correlation between Nf-L values in serum and CSF (See Supplementary Fig. S2 online), a combined analysis of the two fluids is still necessary to obtain a good prediction of the severity of the pathology. However, the CSF Nf-L concentration is not associated with shorter survival contrary to the Nf-L serum levels (Fig. 4c).

### Diagnostic value of multiparametric analysis of CSF and serum biomarkers

Neuroinflammation markers as well as Nf-L were evaluated as diagnostic biomarkers. For this purpose, CSF and serum samples from ALS patients were compared with samples from patients suffering from peripheral inflammatory neuropathies (IPN).

**Figure 5:**
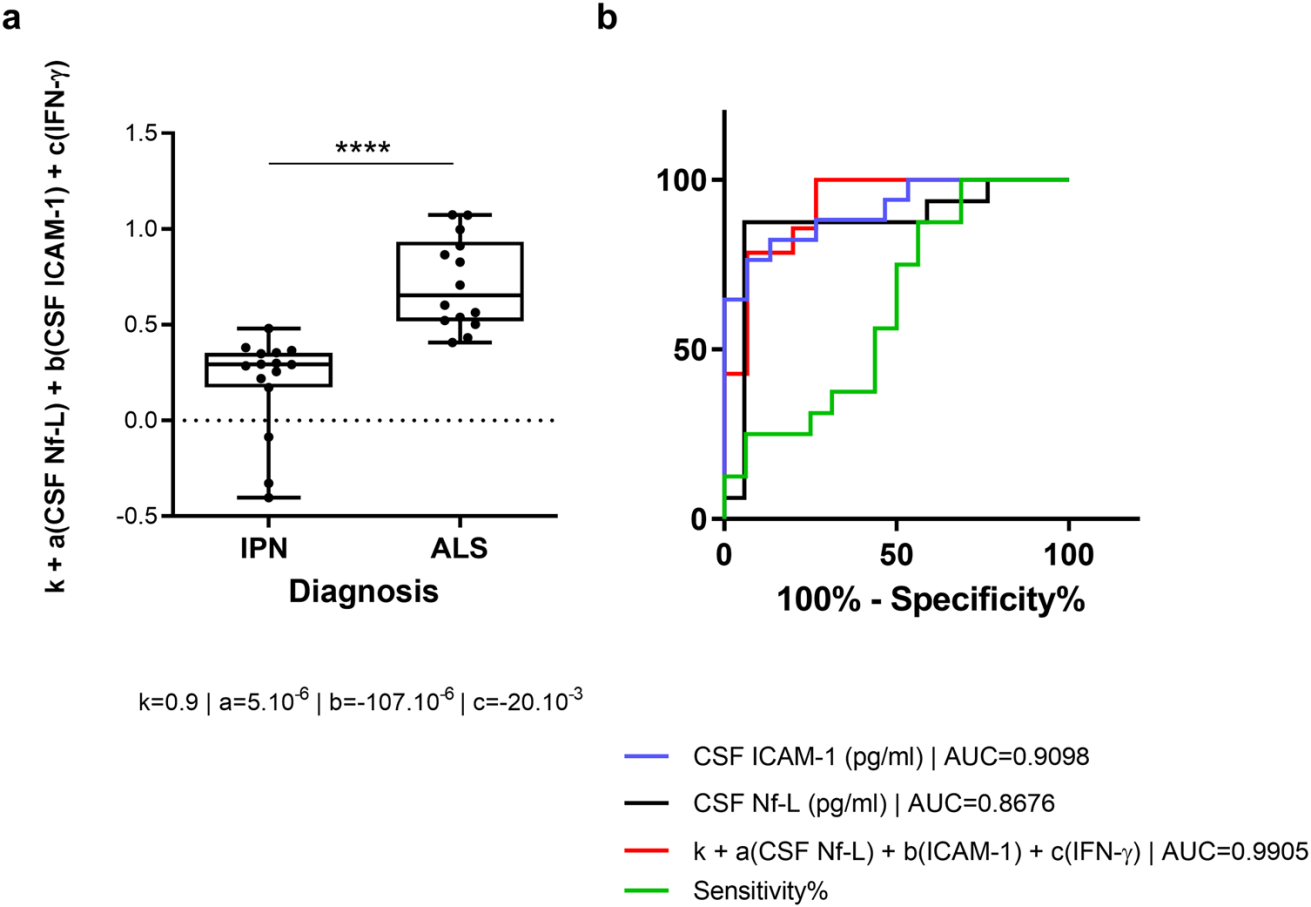
Combination of CSF Nf-L and inflammatory markers as diagnosis biomarker. Diagnosis predictive value of the combination of Nf-L and ICAM-1 CSF levels and IFN-γ serum levels (**a**). ROC Curve analysis CSF Nf-L (black), CSF ICAM-1 (blue), serum IFN-γ (green) and combination the three markers (red) in IPN versus ALS groups (**b**).

Multiple regression analysis of the modified markers between IPN and ALS patients identifies CSF Nf-L and ICAM-1 levels and serum IFN-γ level as the main diagnosis predictors (Table 3). The fitted values obtained using multiple regression model for those three explanatory variables are significantly different between ALS and IPN (Fig. 5a). ROC analysis of ALS and IPN patients provides an Area Under curve (AUC) value of 0.84 for serum Nf-L and an AUC of 0.87 for CSF Nf-L. The optimal cutoff value of 294.2 pg/ml for serum Nf-L provides a specificity of 90.70% for a sensitivity of 74.58% (Likelihood ratio = 8.017) while the optimal cutoff value of 8380 pg/ml for CSF Nf-L provides a specificity of 94.12% for a sensitivity of 87.50% (Likelihood ratio = 14,88).

**Table 3:** Predictive values of the biomarkers on diagnosis by logistic regression analysis.

Predictors	Diagnosis		
	Estimates	CI	*p*
(Intercept)	0.17	− 0.69 to 1.03	0.69
serum Nf-L (pg/ml)	− 0.12 × 10^−3^	− 0.58 × 10^−3^ to 0.34 × 10^−3^	0.58
serum VCAM-1 (pg/ml)	0.51 × 10^−6^	− 0.25 × 10^−6^ to 1.27 × 10^−6^	0.17
serum VEGF (pg/ml)	0.98 × 10^−3^	− 0.25 × 10^−3^ to 2.21 × 10^−3^	0.11
serum IFN-γ (pg/ml)	− 39.91 × 10^−3^	− 76.06 × 10^−3^ to − 3.76 × 10^−3^	**0.03**
CSF Nf-L (pg/ml)	8.37 × 10^−6^	1.38 × 10^−6^ to 15.36 × 10^−6^	**0.02**
CSF VCAM-1 (pg/ml)	63.81 × 10^−6^	− 49.78 × 10^−6^ to 177.41 × 10^−6^	0.25
CSF ICAM-1 (pg/ml)	− 175.70 × 10^−6^	− 0.00035720 to 0.00000581	**0.05**
CSF MCP-1 (pg/ml)	− 0.00011541	− 1571.18 × 10^−6^ to 1340.36 × 10^−6^	0.87
CSF IL-6 (pg/ml)	0.09577709	− 0.22060579 to 0.41215998	0.53
Observations 29			
R^2^/R^2^ Adjusted 0.676/0.522			

Significant *p* values are indicated in bold.

The predictive value of CSF Nf-L levels (AUC = 0.87) regarding ALS diagnosis is increased when combined with CSF ICAM-1 and serum IFN-γ levels (AUC = 0.99) using multiple regression parameters (Fig. 5b).A cutoff value of 0.49 for the fitted values discriminate ALS from IPN patients with a specificity of 100% (78.20–100%) and a sensitivity of 85.71% (57.19–98.22%) with an AUC of 0.99 ± 0.01.

## Discussion

In this study, we used electroluminescent immunoassays to measure Nf-L levels in parallel with neuroinflammation markers in serum and CSF from ALS patients.

CSF level of Nf-L is proposed to be a marker of neuroaxonal damage and neural death in ALS patients^15^. Given the correlation found between the levels in CSF and serum, the concentration of Nf-L in the serum could reflect neurodegenerescence. Indeed, we confirm here the correlation between the ALSFRS slope, reflecting the evolution of the pathology, and the level of Nf-L both in serum and CSF. The ALSFRS score is also correlated with the level of Nf-L only in CSF. A high serum level of Nf-L is associated with lower survival (survival analysis with CSF markers could not be performed due to limited number of data) and CSF as well as serum of Nf-L are elevated in ALS patients compared to controls or IPN patients. Indeed, CSF Nf-L level discriminate ALS from IPN with a 94% sensibility and 81.25% specificity while serum Nf-L level provides a 90.70% sensibility and a 74.58% specificity, in accordance with previous published results^5^.

Nf-L therefore appears to be a biomarker of interest for the severity, survival and diagnosis of ALS. However, this marker is not specific to the disease since there is an increase in Nf-L levels in other neurodegenerative diseases such as Alzheimer’s disease^10^, Multiple Sclerosis^9^ and inflammatory peripheral neuropathies^8^. We therefore analyzed levels of different neuroinflammation markers in ALS and IPN patients in order to increase the predictive value and interest of Nf-L for ALS.

We identified a panel of 3 diagnosis biomarkers for the discrimination between ALS and IPN patients. The model including CSF Nf-L levels combined with CSF ICAM-1 and IFN-γ serum levels reached a 100% specificity and an 85.71% sensitivity.

It should be noted that this model includes markers of different processes involved in the pathophysiology of ALS and IPN, neural cell death (Nf-L), vascular damage (ICAM-1) and peripheral inflammation (IFN-γ).

Evidence of neuroinfammation (reactive astroglia and microglia and infiltrating immune cells) and variations of inflammatory markers in blood and CSF have been described in a mouse model of ALS^16^ as well as in ALS patients^12^. In accordance with previously published studies and meta-analysis^4,14,17^, proinflammatory cytokines and chemokine (IL-6, IL-10, IL-17a, TNF-α, IP-10 and MCP-1) were elevated in the serum of ALS patients when compared to healthy subjects. Although we observe significant variations of several cytokines, chemokines and vascular markers in serum and CSF, there is no correlation between the variations observed in CSF and serum. Cytokine variations in CSF may reflect tissue damage at the central level, while those observed in serum may be representative of the secondary inflammatory response related to the general health of patients. With respect to Nf-L, the increase in serum levels may reflect both the drainage of Nf-L from the CSF into the bloodstream and the release into the circulation related to a peripheral neurodegenerative process. This could explain the fact that the values between the two biological fluids are not very strongly correlated.

ICAM-1 is an adhesion molecule upregulated in endothelial cells in response to inflammatory cytokines (IFN-γ, IL-1β) and regulates activated lymphocytes migration in the CSF. Soluble ICAM-1 is secreted by endothelial cells and PBMC in inflammatory conditions and its level is elevated in vascular and inflammatory pathologies. Elevated levels of VCAM-1 and ICAM-1 have been reported in the CSF of CIDP and GBS patients^18^ explaining the predictive value of this marker in the differential diagnosis of ALS. We here describe an increase in serum level of ICAM-1 in the ALS patients. Previously published data reported an absence of significant difference in serum levels of ICAM-1 between ALS and healthy subjects^19^ despite evidence of vascular alteration in ALS^20^. In view of those results, CSF and serum levels of ICAM-1 need to be investigated as potential biomarkers in larger cohorts.

The decreased level of serum IFN-γ in ALS patients compared to IPN patients reflects the peripheral inflammatory nature of the pathological control group. The levels of serum IFN-γ found in our study are reduced in ALS patients (compared to healthy controls and IPN patients). One previous study found, on the contrary, increased serum levels of IFN-γ in ALS patients compared to healthy subjects^17^ or patients with peripheral neuropathy^21^. The IFN-γ concentrations found using ELISA in these studies were 100 × higher than those reported in our study. Another study using MSD's electrochemiluminescent immunoassay multiplex technology shows on the contrary a decreased of plasma IFN-γ concentrations in ALS patients of similar magnitude to those found in our study^22^. Since the cellular origin of IFN-gamma is primarily T lymphocytes and natural killer cells, a study of immune cells in multicolor cytometry is indicated to complement soluble biomarker studies^23^.

In view of the limited number of patients with CSF sample and the fact that peripheral blood represents more accessible alternative to CSF, we also evaluated the potential of serum markers alone as diagnostic biomarkers for ALS. Specificity and sensitivity regarding ALS diagnosis of serum Nf-L concentration alone (AUC = 0.84; sensitivity = 90.70% (77.86–97.41%); specificity = 74.58% (61.56–85.02%); LR = 8.017) or a model including 3 diagnosis biomarkers (serum Nf-L, serum VCAM-1 and serum IFN-γ) were equivalent. Although there is a significant increase in serum Nf-L concentrations in ALS patients, CSF remains an important tool in the development of biomarkers for ALS diagnosis. In this study, we suggest combining levels of 3 biomarkers from serum (IFN-γ) and CSF (Nf-L and ICAM-1) that could improve diagnostic performance to distinguish ALS from some ALS mimic disorders. We hope to validate these markers on larger cohorts by including, as controls, patients with Progressive Muscular Atrophy and ALS mimic syndromes (motor neuropathies, predominant motor PIDC, MMN, Spinal Muscular Atrophy, Myopathies) in order to evaluate their specificity. A longitudinal patient study will better assess the predictive value of these markers on survival, severity and pathology progression.

## Methods

### Ethical issues

This monocentric study and all experimental protocols were approved by the ethic committee (Comité de Protection des Personnes Sud-Méditerranée 1, April 2014) and registered under the number Eudract 2014-A00786-41. Written informed consent was obtained from all participants and all methods were carried out in accordance with relevant guidelines and regulations.

### Patients

This prospective study included 60 patients with definite ALS according to El Escorial Criteria^24^. We included 20 newly diagnosed patients who underwent lumbar puncture (group 1) and 40 previously diagnosed patients (group 2). Patients are followed in the Referral Center for Neuromuscular Diseases and ALS for a long period of time and until death for some patients allowing analysis of survival prediction. Among the informations collected, the following clinical data were analyzed: sex, age, age at onset of the first symptoms, body mass index, site of onset (either bulbar or spinal), survival after inclusion (months), duration of the disease from onset to inclusion, ALS Functional Rating Score Revisited (ALSFRS-R), diseases progression assessed with the ALSFRS Slope ((48-ALSFRS)/Duration in months). The characteristics of the population are shown in Table 4.

**Table 4:** Summary data of ALS patients.

	Grp 1	Grp 2
	(n = 20)	(n = 40)
**Sex**		
M	17 (85%)	21 (53%)
F	3 (15%)	19 (47%)
Age (years)	66 ± 12	66 ± 11
Age at onset (years)	64 ± 12	63 ± 11
**Site of onset**		
Bulb	3 (15%)	14 (35%)
Spin	17 (85%)	26 (65%)
Survival (months)	10 ± 6.1	35 ± 27
ALSFRS-R	37 ± 7.7	34 ± 7.4
ALSFRS slope	1.2 ± 1.3	0.60 ± 0.44

Healthy controls serum samples were obtained from a previously published cohort^25^. The pathology control group included 43 patients with mainly diagnosed CIDP (40), of whom 17 had a lumbar puncture. (See main characteristics in Supplemental Table 2 online).

### Sample collection and analysis

Samples of CSF on polypropylene tubes, blood on dry tube are rapidly transported, centrifuged (10 min at 1300* g* for serum samples and 5 min at 300 g for CSF samples), aliquoted within 2 h in polypropylene microtubes (Sarstedt 72.692.005) and frozen at − 80 °C until analysis. Nf-L levels were measured with the R-PLEX Human Neurofilament L Antibody Set from MesoScale Discovery (MSD) (F217X, MSD, USA) according to the manufacturer's instructions, Pro-inflammatory cytokines (Il-1β, IL-6, IL-8, IL-17a, IFN-γ, TNF-α), anti-inflammatory cytokine (IL-10), IL-2, chemokines (IP-10, MCP-1, Eotaxin) and vascular markers: VCAM-1, ICAM-1, VEGF) were quantified using the V-PLEX Neuroinflammation Panel 1 Human Kit (K15210D, MSD, USA) according to manufacturer’s instructions.

### Statistical analysis

Analysis was performed using R version 3.03 (R Development Core Team) and GraphPad Prism V6.05 (GraphPad Software, La Jolla, CA, USA). Data are described as Mean ± standart deviation in the tables. Shapiro–Wilk test were used to test for data normality and two-tailed Mann Whitney test were used to test variable differences between groups. Correlations between markers and clinical parameters were evaluated using Pearson correlation analysis. Multiple linear regression was performed using the lm function. Receiver operating characteristic (ROC) Curve analysis were performed between IPN and ALS using ALS as pathological group. Sensitivity [true positive/ (true positive + false negative)] and specificity [true negative/ (true negative + false positive)] were calculated using each concentration value as the cutoff value. Optimal cutoff value was determined as the concentration with the highest positive likelihood ratio [sensitivity/(1-specificity)] in order to obtain the best balance between sensitivity and specificity. Kaplan–Meier survival curves of ALS patients were analyzed using the median markers concentration as cut-off. Significance levels are indicated on graphs (*p < 0.05; **p < 0.01; ***p < 0.001; ****p < 0.0001).

## Supplementary Information

Supplementary Figure 1.

Supplementary Figure 2.

Supplementary Table 1.

Supplementary Table 2.

## Data Availability

Data used in the current study are available from the corresponding author on reasonable request.
