# Supplementary material for: Combination of serum and CSF neurofilament-light and neuroinflammatory biomarkers to evaluate ALS
Source: Sci Rep. 2021 Jan 12;11:703. doi: 10.1038/s41598-020-80370-6 (PMC7803734; doi:10.1038/s41598-020-80370-6)
Supplement: Supplementary file 2 — Supplementary Figure 2. [file 41598_2020_80370_MOESM2_ESM.docx]

**Combination of serum and CSF Neurofilament-Light and neuroinflammatory biomarkers to evaluate ALS**

Alexandre Brodovitch, José Boucraut, Emilien Delmont, Amandine Parlanti, Aude-Marie Grapperon, Shahram Attarian and Annie Verschueren.

**
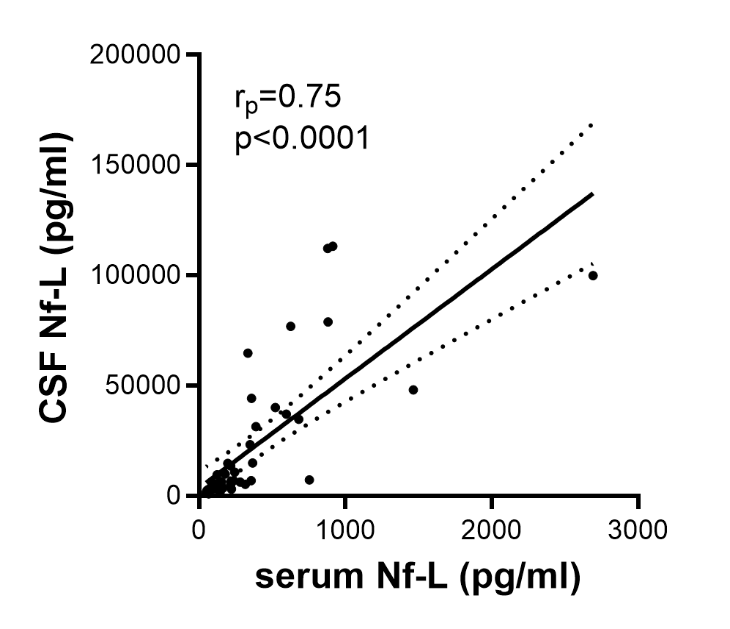
Sup Figure 2.** Correlation between CSF and serum Nf-L levels. Pearson r and significance level are indicated on graph.
